# Two Case Reports of Subacute Thyroiditis after Receiving Vaccine for COVID-19

**DOI:** 10.1155/2022/3180004

**Published:** 2022-04-14

**Authors:** Jan Adelmeyer, Joachim Nils Goebel, Alexander Kauka, Peter Herbert Kann

**Affiliations:** ^1^Philipps-University Marburg, Endocrinology Baldingerstraße, 35043 Marburg, Germany; ^2^General Practice Grossseelheim, Grossseelheim 35274, Germany

## Abstract

The ongoing COVID-19 pandemic, caused by a coronavirus named SARS-CoV-2, has struck the planet with great force. As of December 2019, the virus has made its devasting route across all continents . In January 2022, the World Health Organization (WHO) registered over 5.5 million COVID-19 related deaths. Most of these people had suffered from pneumonia and acute respiratory distress syndrome , and in some cases, extensive damage to all organ systems. To get hold of this pandemic, it was vital to find effective vaccines against it. The two vaccine candidates BNT162b2 (BioNTech/Pfizer) and ChAdOx1 (University of Oxford and AstraZeneca) offer a high level of protection against COVID-19 by providing immunity due to antibody production against the spike protein of SARS-CoV-2. In addition to general side effects, immunological side effects such as subacute thyroiditis can follow the vaccination. This transient inflammatory condition of the thyroid gland is characterized with hyperthyroxinemia, inflammation, pain, and tenderness in the thyroid region, as well as an elevation of serum thyroglobulin concentration. There are only a few reports on the occurrence of this disease after receiving a COVID-19 vaccine. We present two cases of subacute thyroiditis after vaccination with the vaccines BNT162b2 and ChAdOx1 and try to enlighten the problem of immunological phenomena after vaccination. It must be discussed whether cross-reactivity of the spike protein and tissue proteins such as thyroid peroxidase (TPO), an “autoimmune/inflammatory syndrome by adjuvants” (ASIA), or the circulating spike protein itself after vaccination are responsible for the SAT.

## 1. Background

SAT, is an inflammatory disease of the thyroid gland [1-30], which primarily affects women and is manifested by pain and tenderness. Furthermore, patients mention fever, general malaise, myalgia, and arthralgia [10]. These typical clinical symptoms and the presence of elevated free thyroxine (fT4) and free triiodothyronine (fT3) with suppressed thyroid-stimulating hormone (TSH) as well as increased erythrocyte sedimentation rate (ESR) lead to the diagnosis of SAT. Symptoms of thyroid hormone excess may occur in the initial phase, and hypothyroidism affects about 5–10% of patients at annual follow-up [31].

The thyroid gland appears mostly focally hypoechogenic and of normal or enlarged size with no hypervascularity on ultrasound [32]. In addition, 99mTc-pertechnetate uptake is markedly reduced, indicating an inadequate metabolic pathway during inflammation [33].

To date, there is no known definitive cause for this disease. There are some studies that show an association between SAT and HLA-B35 in several ethnic groups, as well as a family-related occurrence [34]. Previous viral infections, such as influenza, coxsackie virus, and mumps virus, have been discussed as potential causes for a long time, but so far this has not been proven with absolute certainty [35]. This theory is partly supported by observing the seasonal occurrence of the SAT from summer to early autumn in some studies [36, 37]. The search for a viral infection is often useless unless there is a therapeutic consequence for the viral disease as such.

Recently, there have been several case reports that showed the occurrence of SAT after infection with SARS-CoV-2 [38]. However, not only could the infection itself trigger SAT, but also the new vaccines are suspected of causing this thyroidal dysfunction [11–26].

We present two additional cases of SAT following vaccination with BNT162b2 (BioNTech/Pfizer) and ChAdOx1 (University of Oxford and AstraZeneca).

## 2. Case Presentation I

A 36-year old woman presented to our endocrinology outpatient clinic in April 2021, four weeks after receiving her second vaccine dose against COVID-19 with BNT162b2 (BioNTech/Pfizer, Comirnaty®). She reported that she had developed neck pain one day after receiving this second dose.

Following symptom onset, she had presented to an ear, nose, and throat doctor. After a laryngoscopy, which found no pathological findings, the doctor performed an ultrasound examination of the thyroid gland, which was assessed to be abnormal. In addition, there was an increased C-reactive protein (CRP), leukocytosis, and an increased thyroglobulin in the external blood tests, so the colleague began a three-day therapy with prednisolone 60 mg/d to treat his suspected diagnosis of SAT. A few hours after starting this treatment the pain disappeared but returned shortly after prednisolone was discontinued. The symptoms worsened over the following weeks, so the woman presented herself in our endocrinology outpatient clinic for further examination and treatment advice. At that time, the patient complained about neck pain, restlessness, as well as fatigue.

She did not have any known preexisting diseases, especially no previous history or family history of thyroid disease or COVID-19. The patient was a cigarette smoker.

### 2.1. Investigation

At examination, she was 170 cm tall and weighed approximately 55 kilograms (body mass index, 19 kg/m^2^). There were no pathological findings regarding heart rate, blood pressure, and temperature. The neck pain could be induced by palpating the thyroid gland. Our initial biochemical blood tests showed a slight leukocytosis as well as moderately increased CRP and significantly increased ESR. In addition, there was a markedly elevated thyroglobulin level with normal fT4, fT3, and TSH indicating an euthyroid state, most likely due to the fact that the patient presented four weeks after the onset of symptoms. No thyroid peroxidase autoantibodies (TPO-Ab), TSH receptor stimulating antibodies (TRAb), or antithyroglobulin antibodies (TgAb) were detected (Table 1).

**Figure 1:**
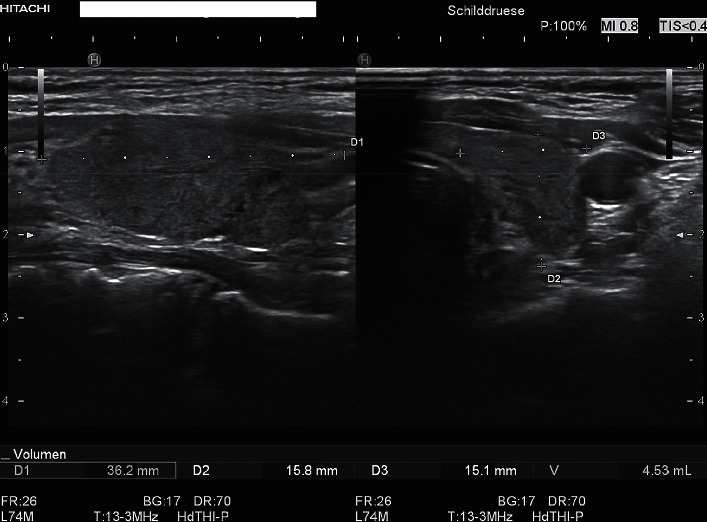
This ultrasound picture shows a hypoechogenic texture with pseudonodular lesions typical for SAT.

Ultrasound examination of the thyroid gland showed a hypoechogenic texture with pseudonodular lesions (Figure 1). The volume was about 18 ml, and some enlarged nonsuspicious lymph nodes were seen. The appearance was typical for SAT, and the pain could be induced by pressure with the ultrasound probe right above the thyroid gland. There was no evidence of a current corona infection in the rapid antigen test.

### 2.2. Treatment

Based on strong evidence suggesting SAT with normal thyroidal function at presentation, we discussed the treatment options with our patient. We offered her therapy with nonsteroidal anti-inflammatory drugs (NSAIDs) or a longer course of prednisolone therapy. She decided on the former, and we advised her to begin a prednisolone therapy if there was no improvement on NSAID with a starting dose of 30 mg/d with tapering the daily dosage by 5 mg every 5–7 days.

### 2.3. Outcome and Follow-Up

During a telephone call two weeks later, the patient reported that the neck pain had worsened while she was taking ibuprofen and that she had developed subfebrile temperatures. Therefore, as recommended, she started prednisolone therapy, which promptly resulted in improvement. She took prednisolone in a tapering scheme over six weeks. The symptoms did not return after stopping the prednisolone, and she has been fine. An intermittent unintentional weight loss of five kilograms was also resolved. The ultrasound examination showed an almost complete recovery. Thyroglobulin decreased by about 75%, and the CRP was normal. Mild leukocytosis may have been explained by smoking. Her thyroid function tests still showed an euthyroid state.

## 3. Case Presentation II

A 65-year old man visited his family doctor in June 2021 because of malaise after receiving his first vaccine against COVID-19 with ChAdOx1 (University of Oxford and AstraZeneca, Vaxzevria®) three days before. Due to an increase in CRP and an increased ESR, the doctor suspected another sigmoid diverticulitis because of several episodes since 2018. The patient also complained of pain in the thyroid region and hoarseness; in secondary findings, a decreased TSH was detected. Therefore, a referral to our endocrinology outpatient clinic was arranged. At the time of the appointment, the local symptoms were decreasing and the patient was fine. There was no history of thyroid disease, neither in the patient nor in their family.

### 3.1. Investigation

The man was in good condition. Blood pressure was normal, and the heart rate was moderately elevated (100 bpm). Due to decreased appetite, he had lost eight kilograms. The thyroid gland was slightly enlarged and palpable without tenderness. Blood examination revealed moderately elevated CRP, normal white blood cell count, mild normochromic normocytic anemia, and elevated ESR. In addition, there was a reduced TSH with normal fT3 and fT4 and increased TgAb, which we assessed as unspecific (Table 1). Other antibodies related to the thyroid gland were negative (TRAb and TPO-Ab).

**Figure 2:**
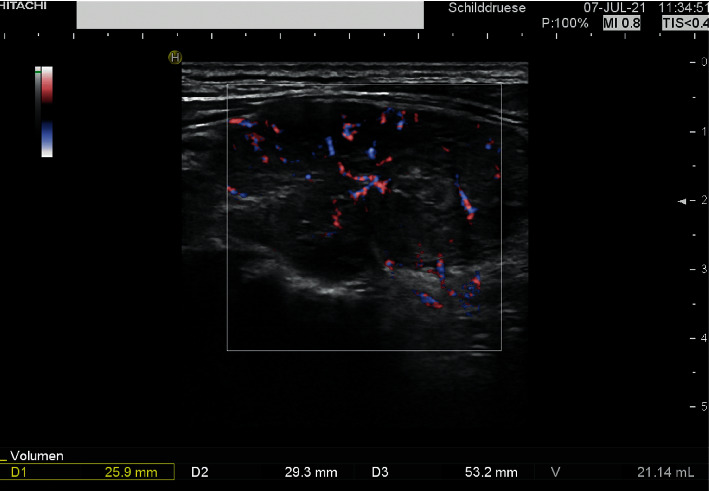
The right lobe of the thyroid gland with a hypoechogenic texture and a diffusely hypoperfused parenchyma.

Ultrasound examination of the thyroid showed a hypoechogenic texture and a diffusely hypoperfused parenchyma. The volume was slightly enlarged (36 ml) and there were some enlarged nonsuspicious lymph nodes (Figure 2). A COVID-19 rapid antigen test was negative.

### 3.2. Treatment

No special treatment was required because of the regressive symptoms.

### 3.3. Outcome and Follow-Up

At the recommended appointment three months later, the patient felt well. Blood tests showed mild anemia but no more signs of inflammation, and the TSH was normalized. Sonographic examination of the thyroid gland showed a reduced volume. There was no tenderness or pressure on the thyroid region.

## 4. Discussion

The current COVID-19 pandemic is causing various medical problems. In addition to the main infections, e.g., lung infection, the infection affects the entire body system. Initial studies by Somasundaram et al. in June 2020 suggested possible damage to the endocrine system by SARS-CoV-2 [39]. Only a few months later, the first reports underscored this suspicion [40]. The thyroid gland could be affected by several mechanisms.

First of all, the virus enters human tissues using the angiotensin-converting enzyme 2 (ACE2) as a cell receptor, which is strongly expressed in thyroid cells and can probably damage the thyroid gland by direct viral cytopathic effects [41, 42]. Early research had shown that there are some homologies between amino acid sequences of, e.g., the spike glycoprotein subunits S1 and S2 from the virus and tissue proteins such as thyroid peroxidase (TPO). This can cause autoimmunity against thyroid cells or exacerbate an existing autoimmunity [43].

In addition, uncontrolled immune responses to the virus in severe cases of COVID-19 should be discussed. This leads to an extensive inflammatory state and an enhanced Th1/Th17 immune response, which results in the release of inflammatory cytokines that are also seen in autoimmune thyroid diseases [44].

Furthermore, the exact pathomechanism of SAT in general is unknown. It is hypothesized that autoreactive T-cells generated by molecular mimicry on thyroid cells promote an autoimmune response against the thyroid gland. Another explanation is that a virus infection results in the presentation of viral products or virus antigens by host cells. As a result, the thyroid cells are recognized as foreign and destroyed by the immune system [45].

But the infection by the virus itself is not only discussed as a cause of SAT. The occurrence of this disease has also been detected after the injection of the new vaccines [11–26]. Recent studies show that the spike protein could be detected in subjects after administration of the mRNA vaccine [30] because both vaccines lead to endogenous production of these spike proteins through different mechanisms to induce immunity against SARS-CoV-2 [6, 7].

In addition, animal experiments have shown that this protein alone can cause cell damage [29]. Since the thyroid cells, as already described, show a high level of expression of the ACE2 receptor, which serves as a cell receptor, direct damage by the protein could be an explanation. However, this theory should be explored in more detail in further studies, and of course, the idea cannot be applied to other vaccinations (e.g., influenza) and the occurrence of SAT.

Another explanation that should be mentioned is the “autoimmune/inflammatory syndrome by adjuvants” known as ASIA. Bragazzi et al. summarized 50 cases of SAT after vaccination that were attributed to this syndrome [28]. Adjuvants are substances that can increase the effectiveness of vaccines and, in some cases, can cause relevant damage by triggering immunological phenomena through activation of B lymphocytes or molecular mimicry [46]. In the case of the vaccine BNT162b2 (BioNTech/Pfizer), lipid nanoparticles (e.g., ALC-0159) have an adjuvant effect in terms of stabilizing the mRNA and transporting it to the target site [47, 48]. In contrast, the vaccine ChAdOx1 (the University of Oxford and AstraZeneca) does not contain any relevant adjuvant substances in the true sense [49] but processed polysorbate 80 (E 433), which is part of AS03, an adjuvant of influenza vaccines [50].

So, ASIA could also be a possible cause of the development of SAT after vaccination against COVID-19. However, ASIA cannot explain the association of SAT with the infection itself.

To our knowledge, there have been publications of 26 cases of SAT following vaccination against SARS-CoV-2 [11–26].

Our cases are in line with the published cases of SAT after COVID-19 vaccination so far (Table 1). The typical clinical presentation with neck pain and malaise, as well as the ultrasound of the thyroid gland and the time since administration (1–3 days), confirm the diagnosis of vaccination-associated SAT. In contrast to most of the cases reported in the literature, our first patient had completely normal thyroid function due to a longer history since the onset of symptoms. Among the 26 cases, there are three others who were euthyroid, and only 62% of the patients had hyperthyroidism. The positive TgAb in our second case was interpreted as unspecific. Nishihara et al. 2019 examined 40 patients in the early phase of SAT and were able to show that 52.5% had positive TgAb, which decreased over time [37]. Three of the 26 cases of vaccine-associated SAT also had positive TgAb.

We must note that although we ruled out corona infection by antigen testing, we did not arrange for further virologic testing for other pathogens. However, there was no evidence of infection.

We would like to point out that, based on the accumulation of case reports, SAT appears to be a possible but rare sequelae of SARS-CoV-2 vaccination that should be considered by practicing physicians. The course of SAT is often mild and disproportionate to the harmfulness of COVID-19 but may be important because of the substantial increase in the global vaccination rate, with more than 9.5 billion doses of vaccine administered [4].

Nevertheless, temporal findings can be no proof of a causal relationship. To date, there have been many reports of SAT following SARS-CoV-2 infection. But a study from a region in northern Italy heavily affected by SARS-CoV-2 could not document a rise in cases of SAT in their outpatient emergency clinic during the pandemic [51]. Likewise, the temporal association of the reported cases of SAT after vaccination against SARS-CoV-2 could still be a coincidence.

Because case reports can only raise suspicion of a causal relationship, studies should compare the incidence of SAT in the weeks after vaccination with an unvaccinated group or with the incidence of SAT before vaccination. In addition, when SAT is diagnosed in temporal association with vaccine use, physicians should make a report to the appropriate pharmacovigilance institution.

## Figures and Tables

**Table 1:** A review of previously published cases of SAT associated with vaccination against SARS-CoV-2. Only SAT cases from each manuscript are listed in this table.

Reference	Gender	Age	Vaccine	Dose	Time from vaccination until onset of symptoms	Symptoms	History of thyroid disease	fT3	fT4	TSH	ESR (mm) (RR < 15)	TgAb	TRAb	TPO-ab
Our case 1	F	36	BNT162b2 (Comirnaty®)	2^nd^	1 day	Neck pain, restlessness, fatigue	None	↔	↔	↔	44	Neg	Neg	Neg
Our case 2	M	65	ChAdOx1 (Vaxzevria®)	1^st^	3 days	Malaise	None	↔	↔	↓	21	Pos	Neg	Neg
Iremli et al. [11]														
Case 1	F	35	PiCoVacc (CoronaVac®)	2^nd^	4 days	Left-side anterior neck pain, fever, fatigue, palpitations	None	↑	↔	↔	53	Neg	Neg	Neg
Case 2	F	34	PiCoVacc (CoronaVac®)	1^st^	4 days	Anterior neck pain, fever, fatigue, palpitations	None	↑	↓	↓	19	Neg	Neg	Neg
Case 3	F	37	PiCoVacc (CoronaVac®)	2^nd^	7 days	Mild anterior neck pain	None	↑	↔	↔	25	Neg	Neg	Neg
Reference	Gender	Age	Vaccine	Dose	Days until symptoms	Symptoms	History of thyroid disease	fT3	fT4	TSH	ESR (mm) (RR < 15)	TgAb	TRAb	TPO-Ab
Bornemann et al. [12]														
Case 1	F	26	ChAdOx1 (Vaxzevria®)	1^st^	14 days	Fever, cervical pain radiated in both ears	None	↑	↔	↔	ND	Neg	Neg	Neg
Case 2	F	49	mRNA-1273 (Spikevax®)	1^st^	14 days	Sore throat with radiation to the ear, headache	None	↔	↔	↔	ND	Neg	Neg	Neg
Patel et al. [13]														
	M	48	ND	2^nd^	7 days	Right neck swelling, throat discomfort, palpitations, fever, weight loss	ND	ND	3.6 ng/dl (no RR)	0.01 mIU/L (no RR)	ND	ND	ND	ND
Reference	Gender	Age	Vaccine	Dose	Days until symptoms	Symptoms	History of thyroid disease	fT3	fT4	TSH	ESR (mm) (RR < 15)	TgAb	TRAb	TPO-ab
Saygili et al. [14]														
	F	38	PiCoVacc (CoronaVac®)	2^nd^	14 days	Neck swelling, pain, fatigue, loss of appetite, sweating	None	↑	↔	↓	78	Neg	ND	Neg
	F	34	BBV152 (COVAXIN®)	1^st^	5–7 days	Fever, palpitation, anterior neck pain	None	↑	↑	↓	60	ND	ND	ND
Chatzi et al. [15]														
	F	35	BNT162b2 (Comirnaty®)	1^st^	12 days	Neck pain, fatigue, palpations	ND	ND	↑	↓	75	Neg	Neg	Neg
	F	32	BNT162b2 (Comirnaty®)	2^nd^	4 days	Neck pain, fatigue	ND	ND	↔	↓	40	Neg	Neg	Neg
Reference	Gender	Age	Vaccine	Dose	Days until symptoms	Symptoms	History of thyroid disease	fT3	fT4	TSH	ESR (mm) (RR < 15)	TgAb	TRAb	TPO-ab
Kyriacou et al. 10/21 [16]														
	F	40	BNT162b2 (Comirnaty ®)	2^nd^	12 hours	Malaise, anterior neck pain,	None	ND	↑	↓	67	Pos	Neg	Neg
Siolos et al. [17]														
	F	51	BNT162b2 (Corminaty®)	1^st^	4 days	Nausea, mild anterior neck pain, fever	None	↔	↑	↓	103	Neg	Neg	Neg
Reference	Gender	Age	Vaccine	Dose	Days until symptoms	Symptoms	History of thyroid disease	fT3	fT4	TSH	ESR (mm) (RR < 15)	TgAb	TRAb	TPO-ab
Jeeyavudeen et al. [18]														
	F	ND	BNT162b2 (Comirnaty®)	2^nd^	14 days	Painful swelling, poor sleep, night sweats, hyperdefecation, weight loss	None	↑	↑	↓	ND	Neg	ND	Neg
Plaza-Enriquez et al. [19]														
	F	42	mRNA-1273 (Spikevax®)	2^nd^	5–6 days	Earache radiating down to the neck and jaw,	None	↑	↔	↓	81	ND	ND	Neg
Reference	Gender	Age	Vaccine	Dose	Days until symptoms	Symptoms	History of thyroid disease	fT3	fT4	TSH	ESR (mm) (RR < 15)	TgAb	TRAb	TPO-ab
Khan et al. [20]														
	F	42	BNT162b2 (Comirnaty®)	2^nd^	4 days	Fever, palpitations, painful left-sided neck swelling	ND	ND	↑	↓	60	ND	Neg	Neg
Sözen et al. [21]														
	M	41	BNT162b2 (Comirnaty®)	2^nd^	8 days	Anterior neck pain, fatigue, palpitation	None	↑	↑	↓	32	Neg	Neg	Neg
	F	40	BNT162b2 (Comirnaty®)	1^st^	6 days	Neck pain, palpitation, sweating	None	↔	↔	↓	34	Pos	Neg	Neg
	M	40	BNT162b2 (Comirnaty®)	1^st^	4 days	Neck pain, nervousness, fatigue	None	↔	↔	↔	15	Neg	ND	Neg
Reference	Gender	Age	Vaccine	Dose	Days until symptoms	Symptoms	History of thyroid disease	fT3	fT4	TSH	ESR (mm) (RR < 15)	TgAb	TRAb	TPO-Ab
	F	26	BNT162b2 (Comirnaty®)	2^nd^	6 days	Neck pain	None	↑	↑	↓	34	Pos	Neg	Pos
	F	44	BNT162b2 (Comirnaty®)	2^nd^	9 days	Neck pain, headache, palpitation, sweating, tremor	Hashimoto thyroiditis	↔	↔	↓	44	Neg	Neg	Pos
Schimmel et al. [22]														
	F	57	BNT162b2 (Comirnaty®)	2^nd^	1 day	Anterior neck pain, swelling	None	↔	↔	↓	ND	Neg	Neg	Neg
Franquemont et al. [23]														
	F	42	BNT162b2 (Comirnaty®)	1s^t^	5 days	Sore throat, palpitations	None	11.8 (no RR)	4.58 (no RR)	<0.01 (no RR)	62	Neg	Neg	Neg
Reference	Gender	Age	Vaccine	Dose	Days until symptoms	Symptoms	History of thyroid disease	fT3	fT4	TSH	ESR (mm) (RR < 15)	TgAb	TRAb	TPO-ab
Oyibo et al. [24]														
	F	55	ChAdOx1 (Vaxzevria®)	1^st^	21 days	Headache, sore throat, generalized aches, palpitations	None	ND	↑	↓	51	ND	ND	Neg
Sahin Tekin et al. [25]														
	M	67	PiCoVacc (CoronaVac®)	2^nd^	19 days	Fever, mild neck pain,	None	↑	↑	↓	67	Neg	Neg	Neg
Reference	Gender	Age	Vaccine	Dose	Days until symptoms	Symptoms	History of thyroid disease	fT3	fT4	TSH	ESR (mm) (RR < 15)	TgAb	TRAb	TPO-ab
Pla Peris et al. [26]														
	M	67	mRNA-1273 (Spikevax®)	1^st^	10–14 days	Neck pain radiating to the ears, asthenia, mild fever, tachykardia	None	ND	↑	↓	60	Neg	Neg	Pos
	M	47	BNT162b2 (Comirnaty®)	1^st^	10–14 days	Neck pain radiating to the ears, asthenia, mild fever, tachykardia	None	ND	↔	↓	70	Neg	Neg	Neg

Note: the pathological values are printed in bold. fT3: free triiodothyronine; fT4: free thyroxine; TSH: thyroid-stimulating hormone; ESR: erythrocyte sedimentation rate; TgAb: antithyroglobulin antibody; TRAb, TSH receptor antibodies; TPO-Ab: thyroid peroxidase antibody; pos: positive; neg: negative; ND: no data; RR : reference range; ↔: normal; ↑: increased; ↓: decreased.

## Data Availability

The data used to support the findings of this study are included within the article.

## Additional Points

Subacute thyroiditis (SAT) is a temporary inflammatory disease of the thyroid gland that is probably triggered by multiple viral infections with a genetic predisposition to HLA-B35. In the past, the occurrence of SAT has been described, for example, after influenza vaccination. Recently, several reports have described the appearance of SAT as being associated with the injection of the new COVID-19 vaccines. Cross-reactivity between the spike protein and tissue proteins of the thyroid gland, ASIA, or the circulating spike protein as a cause for SAT after vaccination should be discussed.
